# Sirt3 Rescues *Porphyromonas gingivalis*‐Impaired Cementogenesis via SOD2 Deacetylation

**DOI:** 10.1111/cpr.70022

**Published:** 2025-03-11

**Authors:** Xin Huang, Huiqing Gou, Jirong Xie, Yonglin Guo, Yifei Deng, Yan Xu, Zhengguo Cao

**Affiliations:** ^1^ State Key Laboratory of Oral & Maxillofacial Reconstruction and Regeneration, Key Laboratory of Oral Biomedicine Ministry of Education, Hubei Key Laboratory of Stomatology School & Hospital of Stomatology, Wuhan University Wuhan China; ^2^ Department of Periodontology School & Hospital of Stomatology, Wuhan University Wuhan China; ^3^ State Key Laboratory Cultivation Base of Research, Prevention and Treatment for Oral Diseases Nanjing Medical University Nanjing China; ^4^ Department of Periodontology Affiliated Hospital of Stomatology, Nanjing Medical University Nanjing China; ^5^ Jiangsu Province Engineering Research Center of Stomatological Translational Medicine Nanjing China

**Keywords:** acetylation, cementogenesis, cementum, *porphyromonas gingivalis*, sirtuin3, superoxide dismutase

## Abstract

The keystone pathogen *Porphyromonas gingivalis* (P.g.) is responsible for cementum resorption in periodontitis; however, the mechanism involved in it remains unclear. Sirtuin 3 (Sirt3) is a NAD^+^‐dependent protein deacetylase contributing to mitochondrial homeostasis and various cell functions. In this study, the expression of Sirt3 in cementoblasts was found to be increased during cementoblast mineralisation and cementum development, while it decreased gradually under P.g. infection in a multiplicity of infection‐dependent manner. Compared with wild type mice, the Sirt3 knockout mice showed less cellular cementum and lower mineralisation capacity with decreased expression of Runx2 and OCN in cementoblasts. Sirt3 inhibition by 3‐TYP or Sirt3 silencing by lentivirus infection both confirmed the impaired cementogenesis. Conversely, honokiol (HKL) was simulated to bind Sirt3 and was applied to activate Sirt3 in cementoblasts. HKL‐mediated Sirt3 activation facilitated cementoblast mineralisation and rescued P.g.‐suppressed cementoblast mineralisation markedly. Superoxide dismutase 2 (SOD2), the downstream molecule of Sirt3, showed a similar expression pattern to Sirt3 under different conditions. Silencing of SOD2 was demonstrated to restrain cementoblast mineralisation. The pan acetylation was detected to decrease under Sirt3‐upregulating conditions and increase under Sirt3‐downregulating conditions. The binding of Sirt3 and SOD2 in cementoblasts was also verified. Furthermore, SOD2 acetylation and specific SOD2‐K68 acetylation were found to be upregulated under P.g. or Sirt3 silencing conditions and downregulated by HKL stimulation. Moreover, K68Q mutation simulating acetylation decreased cementoblast mineralisation, while K68R mutation simulating deacetylation increased it. Altogether, Sirt3 deacetylates SOD2 via K68 to orchestrate P.g.‐perturbed cementogenesis, and HKL is a Sirt3‐targeted treatment candidate.

## Graphical abstract

Sirt3 deacetylated SOD2 via K68 to orchestrate P.g.‐impaired cementogenesis. Reestablishment of the Sirt3/SOD2 axis by honokiol reduced SOD2 acetylation and facilitated cementogenesis.

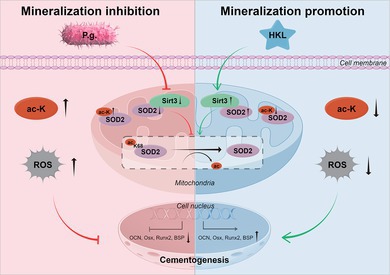

## 1 Introduction

Cementum is the mineralised tissue covering the tooth roots and serves to anchor the tooth in alveolar bone by the collagen fibres embedded in it [1]. In view of the role of cementum in tooth stability and dental health, the maintenance and neo‐genesis of cementum were identified as a critical part for functional periodontal homeostasis [2, 3]. However, cementum resorption can be associated with periodontitis [4], the chronic inflammatory disease of periodontal tissues, which is dominated by the keystone pathogen *Porphyromonas gingivalis* (P.g.) [5, 6]. Though our previous work demonstrated the suppressive role of P.g. in cementogenesis and cementoblast (CB) mineralisation [7, 8], the biology and mechanism involved in P.g.‐disturbed CB mineralisation remain unclear.

Sirtuins are a family of nicotinamide adenine dinucleotide (NAD^+^)‐dependent protein deacetylases that contain seven members (Sirt1–7) [9]. Among them, sirtuin 3 (Sirt3) is located in mitochondria and cytoplasm, with a role in regulating mitochondrial homeostasis mainly via the deacetylation capacity [10]. Given the diversity of the deacetylation targets, Sirt3 is involved in the regulation of different cell functions including proliferation, death, metabolism and differentiation [11, 12, 13]. Further, Sirt3 is well‐documented to be related to various diseases, such as ageing, inflammation and cancer [9, 14]. Sirt3 is a positive regulator of osteogenesis and bone formation. It is demonstrated to be a potential target for treating bone diseases [15]. In terms of periodontitis, Sirt3 deficiency is demonstrated to aggravate age‐associated periodontal bone destruction and compromise the irisin‐protected bone loss [16, 17]. As with other hard periodontal tissue, cementum shares similarities with bone [18]; however, the regulatory relationship between Sirt3 and cementogenesis under P.g. infection has not been reported.

The superoxide dismutase (SOD) family comprises three members (SOD1–3), among which the mitochondrial matrix‐located SOD2 encodes manganese superoxide dismutase (Mn‐SOD) to modulate oxidative stress in cells [19]. Besides ageing, inflammation and cardiovascular diseases, SOD2 is also verified to be a protective regulator against osteoporosis [20, 21, 22]. Moreover, SOD2 is proved to be the deacetylation target of Sirt3 in orchestrating bone homeostasis. Through controlling reactive oxygen species (ROS), Sirt3/SOD2 axis activation or deficiency results in osteogenesis or osteoclastogenesis, respectively [13, 23]. Nevertheless, the changes of SOD2 in CBs in response to P.g. stimulation and the regulatory role of SOD2 in CB mineralisation have not been uncovered.

In this study, the expression pattern of Sirt3 in CBs during mineralisation or P.g. infection was studied. By means of the Sirt3^−/−^ mice, chemical inhibitor and gene silencing methods, the role of Sirt3 in cementogenesis was elucidated. Further, a natural drug activating Sirt3 was applied to investigate the effect of Sirt3 upregulation on CB mineralisation under P.g. infection. The involvement of the Sirt3/SOD2 axis in P.g.‐inhibited cementogenesis was also uncovered. Moreover, the deacetylation site of SOD2 by Sirt3 was explored. Altogether, this study may contribute to cementum reparation perturbed by P.g.

## 2 Materials and Methods

### 2.1 Cell Culture

The immortalised mouse CB cell line OCCM‐30 was gifted by Dr. M.J. Somerman. OCCM‐30 cells were maintained in high‐glucose Dulbecco's modified Eagle medium (DMEM; Hyclone, USA) containing 10% fetal bovine serum (Gibco, USA) at 37°C with 5% CO_2_. Osteogenic induction medium (OIM) was prepared with complete medium, 10 mM sodium β‐glycerophosphate, 50 μg/mL vitamin C and 10 nM dexamethasone (Sigma, USA).

### 2.2 *Porphyromonas gingivalis* Culture

P.g. (ATCC 33277) was cultured in trypticase soy broth containing 5 μg/mL hemin, 1 μg/mL vitamin K1 and 0.1% yeast extract (Sigma) at 37°C in an anaerobic environment (10% CO_2_, 10% H_2_, 80% N_2_). P.g. concentration was determined according to the OD value at 600 nm (1 OD = 10^9^ P.g./mL).

### 2.3 Heatmap Analysis

Total RNA of OIM‐cultured (7 days) OCCM‐30 cells and their control (CON) cells was extracted by TRIzol (TaKaRa, Japan) for sequencing. Quality control, NGS and data analysis were conducted by ANOROAD (Beijing, China). Mineralisation‐associated genes and sirtuin family genes (Sirt1–7) were selected. The RPKM (reads per kilobase per million mapped reads) value was set as the indicator of gene expression. Heatmaps were generated by TBtools (https://github.com/CJ‐Chen/TBtools/releases).

### 2.4 Cytotoxicity Test

3‐TYP and honokiol (HKL) were purchased from MedChemExpress (Monmouth Junction, NJ, USA) and used to inhibit or activate the expression of Sirt3 in OCCM‐30 cells, respectively. Cytotoxicity was evaluated by cell counting kit‐8 (CCK‐8; Life‐iLab, Shanghai, China). Briefly, cells were seeded in 96‐well plates with a density of 2000/well. After stimulation with different concentrations of drugs for different days, cells in each well were incubated with 100 μL of CCK‐8 working solution for 1 h. Absorbance at 450 nm was documented.

### 2.5 Quantitative Real‐Time Polymerase Chain Reaction (RT‐qPCR)

RNA was extracted using TRIzol (TaKaRa). 500 ng RNA of each sample was reversely transcribed into cDNA by the PrimeScript RT Reagent Kit (TaKaRa). SYBR qPCR Master Mix (Vazyme, China), 10 μM primers (Sangon, China) and cDNA were applied to conduct qPCR by Bio‐Rad CFX96. The thermocycling method, with initial denaturation at 95°C for 30 s, 40 cycles at 95°C for 10 s, 62°C for 34 s and extension at 72°C for 30 s, was used. Values were normalised to *β‐actin*. The 2−ΔΔCT^−ΔΔCt^ method was used for quantification. Primers are listed in Table S1.

### 2.6 Western Blot (Immunoblotting, IB)

Proteins were extracted by M‐PER mammalian protein extraction reagent (Thermo Scientific, USA). 20 μg of protein from each sample was loaded onto a 4%–12% FuturePAGE precast gel (ACE, China) for electrophoresis. Proteins were then transferred onto polyvinylidene difluoride (PVDF) membranes (Millipore, USA). After 5% milk blocking for 2 h at room temperature, membranes were incubated with primary antibodies overnight at 4°C. Then, membranes were incubated with horseradish peroxidase‐conjugated goat anti‐rabbit/mouse secondary antibodies for 1 h at room temperature. Bands were visualised using Super ECL Detection Reagent (BioPM, China) by the Odyssey LI‐COR scanner. Information about the antibodies is listed in Table S2.

### 2.7 ALP and Alizarin Red Staining

After osteogenic induction, cells were fixed with 4% paraformaldehyde for 10 min. ALP staining (7 days) was conducted by the BCIP/NBT ALP Colour Development Kit (Beyotime) according to the manufacturer's instructions. Images were taken and quantified by Image J. Mineralisation capacity (10, 14 days) was detected by staining the cells with Alizarin Red S Solution (Oricell, China) for 15 min. Mineral nodules were pictured and dissolved with 10% cetylpyridinium chloride (Yuanye, China). Absorbance at 562 nm was quantified.

### 2.8 Co‐Immunoprecipitation (Co‐IP)

The interacting proteins of Sirt3 with an interaction score > 0.9 were mapped by the STRING database. The IP reagent kit was purchased from Absin (China). Extracted proteins were incubated with Protein A/G agarose beads for 1 h to reduce nonspecific binding and then incubated with Sirt3 or SOD2 antibody (3 μg) overnight at 4°C. Protein A/G agarose beads were added and incubated for 3 h at 4°C on a shaker to pull down immune complexes. The proteins were finally eluted and denatured with SDS loading buffer (Beyotime, China). Interacting proteins were detected by IB (western blot). Information about the antibodies is listed in Table S2.

### 2.9 Immunofluorescence (IF)

OCCM‐30 cells were infected with different multiplicity of infection (MOI) values of P.g. for 1 day. After PBS washing, fixation, 0.5% Triton X‐100 (Biofroxx, GER) permeabilisation and goat serum blocking for 1 h, cells were incubated with primary antibodies of P.g. and Sirt3 together overnight at 4°C. Then, the cells were incubated with secondary antibodies conjugated with FITC or Cy3 for 1 h, and also anti‐fade mounting medium with DAPI (Beyotime) for 5 min. Fluorescence was observed with a microscope. Information about the antibodies is listed in Table S3.

### 2.10 Lentivirus Infection

Lentiviruses containing a Sirt3‐ and SOD2‐specific short hairpin RNAs as well as the control were constructed by JTSBIO g Ltd. (Wuhan, China). OCCM‐30 cells were transfected with the lentivirus (MOI = 50) with 5 ng/mL polybrene (JTSBIO Co. Ltd) for 1 day. Puromycin (2 μg/mL; Biosharp, China) was used to screen the transfected cells. Green fluorescence was used to evaluate the transfection efficiency. Targeting sequences of Sirt3 and SOD2 are listed in Table S4.

### 2.11 Histological Staining

This study conformed to the updated ARRIVE 2.0 guidelines for preclinical animal studies. All animal experiments were approved by the Ethics Committee of the School and Hospital of Stomatology, Wuhan University and the Experimental Animal Welfare and Ethics Committee of Nanjing Medical University (IACUC‐2006020). Mice were housed under controlled conditions (55% ± 5% humidity; 22°C ± 2°C; 12‐h light/dark cycle). To study the expression of Sirt3 in CBs during cementum development, mandibles of C57BL/6 mice (*n* = 5/group; male) aged 3, 6 weeks and 6 months were collected. To study the regulatory effect of Sirt3 on cementum formation in vivo, samples of *Sirt3* knockout (KO) mice (*Sirt3* ^−/−^) and their littermate control wild‐type (WT) mice (*n* = 6/group; 8 w, 22 ± 1.5 g, male) were also collected. Samples were fixed, decalcified, paraffin embedded and cut into 4 μm sections. The difference of first molars was observed. Haematoxylin and eosin (HE) staining was performed to evaluate the cementum area. Immunohistochemistry (IHC) was performed using the UltraSensitive SP IHC Kit (MXB Biotech, China) following the manufacturer's instructions. Information about the antibodies used in IHC is listed in Table S3. Images were taken by a microscope and quantified by Image J.

### 2.12 Genotype Identification

*Sirt3* KO mice generated in a C57BL/6J background were constructed by Cyagen Biotechnology Co. Ltd. DNA was extracted from the tails of genetically modified mice according to the One Step Mouse Genotyping Kit (Vazyme, China), and then agarose gel electrophoresis was used for genotyping identification. Primers for *Sirt3* KO PCR: F1 CAGTCAGTGACATCTTGGCTCTAC; R1 CAGCCCAGCCTTATGTTCCTTTAC and R2 CAAAGCAAATCTCAGTGTTGCAGC.

### 2.13 Micro‐CT

Micro‐CT scanning (vivaCT80, SCANCO medical, 55 kVp, 144 μA, 15.6 μm, Switzerland) was performed on the maxillas and mandibles of the above‐mentioned *Sirt3* KO mice and WT controls. Subsequently, the maxillary and mandibular two‐dimensional images were reconstructed using SkyScan (Bruker) software to observe and analyse the thickness of the cementum–dentin complex at the apical region of the first molar teeth.

### 2.14 Sem

Mandibular first molars from Sirt3 KO mice and WT mice mentioned above were polished by successively finer grades of SiC abrasive paper (# 800, # 1500, # 2000, # 3000, # 5000, # 7000) sagittally. After gold spraying, the samples were observed and photographed by SEM (Zeiss SIGMA, GER).

### 2.15 SOD2 Mutation and Plasmid Transfection

Conservation of SOD2‐K68 from different species was confirmed by the NCBI database. Mouse SOD2 cDNA was cloned into pcDNA3.1 by the BamHI/XhoI strategy. The 68‐K (lysine) of SOD2 was mutated to Q (glutamine) to simulate acetylation and mutated to R (arginine) to simulate deacetylation. The SOD2‐WT, SOD2‐K68Q and SOD2‐K68R plasmids were constructed and sequenced by GeneCreate (Wuhan, China). The plasmids were transfected into OCCM‐30 cells using TurboFect (Thermo Scientific) according to the manufacturer's protocol. OIM was added 1 day after the transfection.

### 2.16 ROS Detection

Intracellular ROS was detected by a ROS assay kit (Beyotime) using the non‐fluorescent probe DCFH‐DA that could be oxidised to produce fluorescent DCF. Briefly, DCFH‐DA was diluted in serum‐free medium at 1:1000 to a final concentration of 10 μM and then incubated with cells attached in 96‐well plates for 20 min at 37°C. After serum‐free medium washing, the ROS level indicated by fluorescence intensity at 488 nm excitation and 525 nm emission was measured.

### 2.17 Molecular Docking

The CID (72303) of HKL was obtained from PubChem Compound. The PDB code (3GLS) of Sirt3 was downloaded from the RCSB PDB. Molecular docking studies were performed by Autodock Vina 1.2.2 on a simplified molecular docking platform (https://www.home‐for‐researchers.com/#/). The result with the lowest binding energy value (highest binding activity) was chosen.

### 2.18 Statistical Analysis

Data were presented as mean ± SD of at least three independent experiments and analysed using GraphPad Prism 8. The Shapiro–Wilk test was used to determine normal distribution. The Student's *t*‐test was used to assess differences between two groups. One‐way ANOVA was used to assess differences between multiple groups, followed by Bonferroni correction. Significance was defined as *p < 0.05, **p < 0.01 and ***p < 0.001. *p* > 0.05 was not considered significant (ns).

## 3 Results

### 3.1 Sirt3 Increases During Cementogenesis While Decreases Under *Porphyromonas gingivalis* Infection in Cementoblasts

**FIGURE 1:**
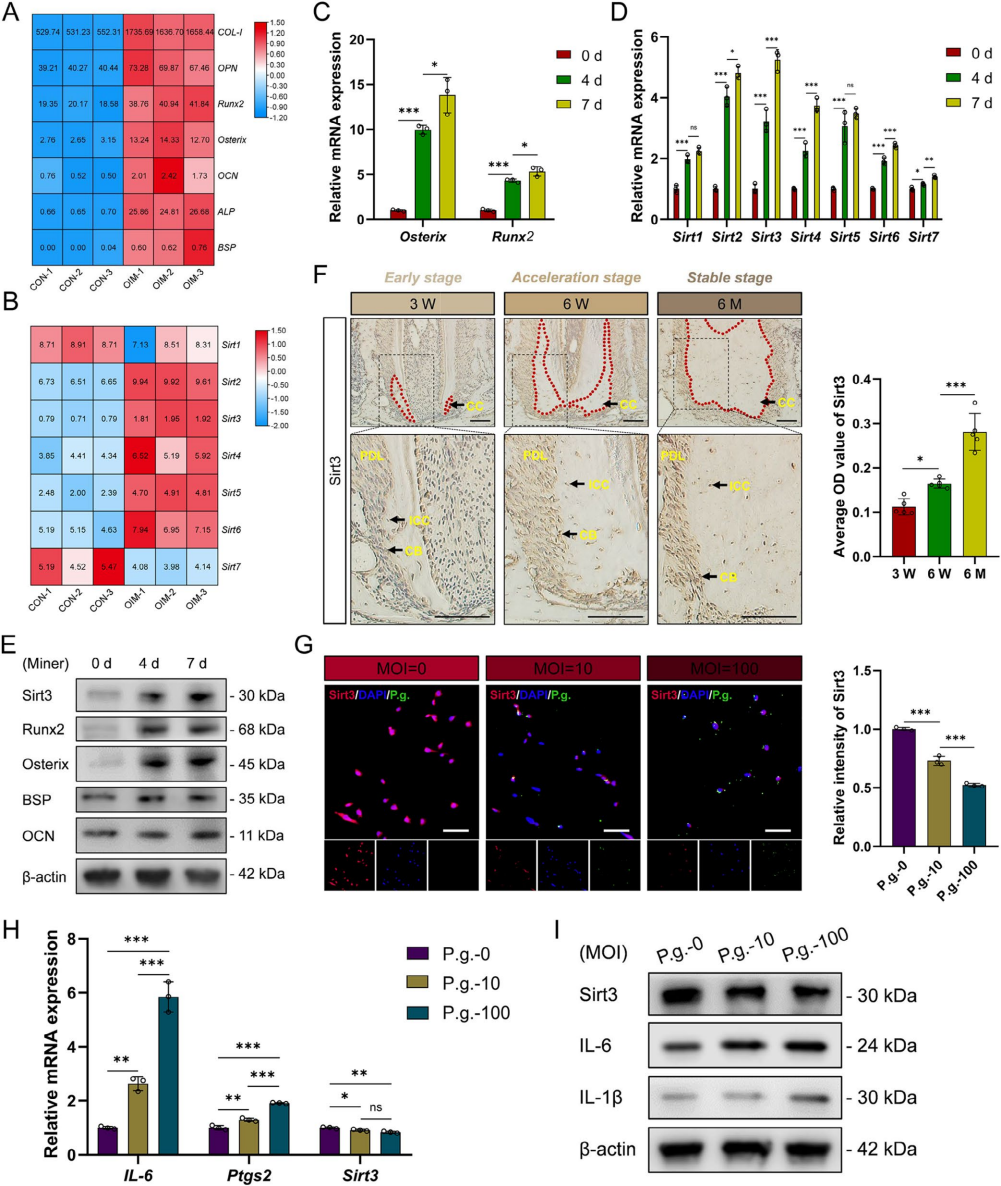
Sirt3 in cementoblasts increases during cementogenesis while decreases under P.g. infection. Heatmaps of the expression of mineralisation‐related markers (A) and sirtuin family members (B) in OCCM‐30 cells induced for 0 (CON) and 7 days (OIM) were analysed from mRNA sequencing results. mRNA expression of Osterix, Runx2 (C) and Sirt1–7 (D) in OCCM‐30 cells induced for 0, 4 and 7 days was verified by RT‐qPCR. Protein expression of Sirt3 and mineralisation‐related markers was detected by western blot (E). Sirt3 expression in cementoblasts of the mandibular first molars from mice aged 3, 6 weeks and 3 months were examined by IHC (F). Sirt3 expression (red) in OCCM‐30 cells infected with different MOIs (0, 10, 100) of P.g. (Green) for 1 day was observed by IF (G). The expression of Sirt3 and inflammation‐related markers in OCCM‐30 cells infected with different MOIs (0, 10, 100) of P.g. for 1 day was detected by RT‐qPCR (H) and western blot (I). CON, control; IF, immunofluorescence; IHC, immunohistochemistry; MOI, multiplicity of infection; OIM, osteogenic induction medium; P.g., *porphyromonas gingivalis* ; RT‐qPCR, quantitative real‐time polymerase chain reaction. *p < 0.05, **p < 0.01 and ***p < 0.001. *p* > 0.05 was considered not significant (ns).

By mRNA sequencing, it was found that mineralisation‐related markers in OCCM‐30 cells increased after 7 days of induction (Figure 1A). Accordingly, most of the sirtuin family genes increased in the OIM group (Figure 1B). To verify the results, OCCM‐30 cells were induced for different days. The results showed that the mRNA expression of all sirtuins was upregulated gradually along with the mineralisation time; of note, the variation trend of Sirt3 is the most significant (Figure 1C,D). Also, the positive correlation between Sirt3 protein expression and mineralisation in OCCM‐30 cells was demonstrated (Figure 1E). To confirm the above findings in vivo, mice aged 3, 6 weeks and 6 months were applied. The expression of Sirt3 in CBs of mandibular first molars was found to be increased during cementum deposition at different developmental stages (Figure 1F).

Then, OCCM‐30 cells were exposed to different MOIs of P.g., a threat for CB mineralisation. Contrary to the expression of inflammatory cytokines, Sirt3 expression was downregulated under P.g. infection in a MOI‐dependent manner (Figure 1G–I). In all, these results revealed a high correlation between Sirt3 and cementum mineralisation.

### 3.2 Sirt3 Deficiency Impairs Cementogenesis In Vitro and In Vivo

**FIGURE 2:**
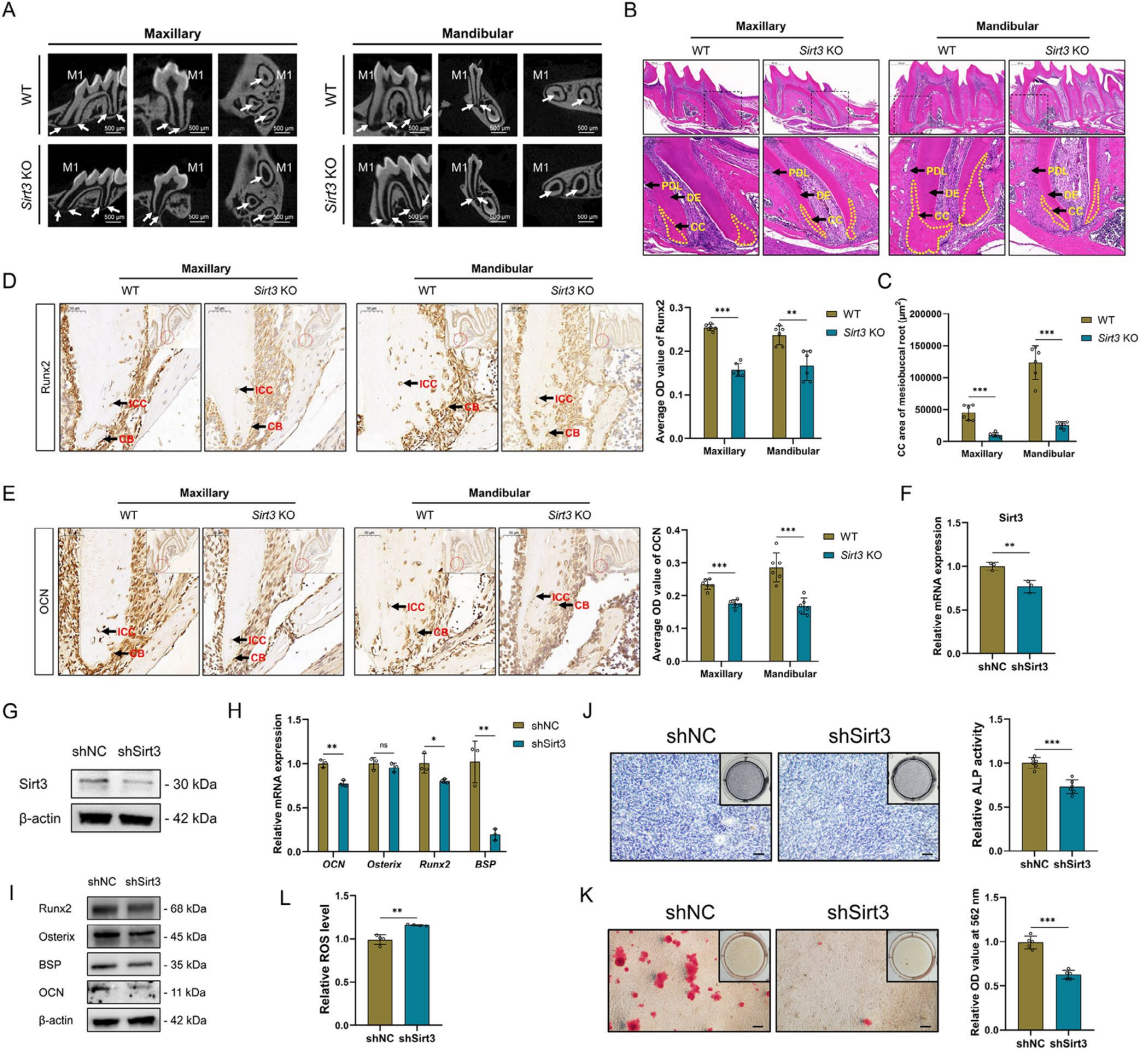
Sirt3 deficiency impairs cementogenesis in vitro and in vivo. Gross observation of cementum–dentin complex of the first molars of WT mice and *Sirt3* KO mice by micro‐CT (A). HE staining (B) and quantification (C) of cellular cementum of the first molars of WT mice and *Sirt3* KO mice. The expression of Runx2 (D) and OCN (E) in cementoblasts of the first molars of WT mice and *Sirt3* KO mice. The expression of Sirt3 in Sirt3‐silenced OCCM‐30 cells (shSirt3) and the control cells (shNC) was verified by RT‐qPCR (F) and western blot (G). The mRNA and protein expression of mineralisation‐related markers in shNC and shSirt3 cells induced for 2 days was detected by RT‐qPCR (H) and western blot (I), respectively. ALP activity of shNC and shSirt3 cells induced for 7 days was measured by ALP staining (J). Mineralised nodule formation capacity of shNC and shSirt3 cells induced for 10 days was measured by ARS (K). Intracellular ROS level in shNC and shSirt3 cells was determined by a ROS assay kit (L). ALP, alkaline phosphatase; ARS, alizarin red staining; KO, knockout; M1, first molar; ROS, reactive oxygen species; WT, wild type. *p < 0.05, **p < 0.01 and ***p < 0.001. *p* > 0.05 was considered not significant (ns).

To determine the essential role of Sirt3 in cementogenesis, the cementum of the first molars from *Sirt3* KO and WT mice was compared (Figure S1). By micro‐CT, a significant reduction in the cementum–dentin complex and a larger root canal width in *Sirt3* KO mice was found when compared with WT mice (Figure 2A). Further, SEM (Figure S2) and HE staining results (Figure 2B,C) both verified that cellular cementum (CC) formation in mice was critically impaired, as well as dentin (DE) formation, when Sirt3 was knocked out. Accordingly, a lower mineralisation capacity of CBs and immature cementocytes (ICCs) in *Sirt3* KO mice with decreased expression of Runx2 and OCN was demonstrated (Figure 2D,E).

Then, chemical inhibitor 3‐TYP with a non‐cytotoxic concentration was used to suppress Sirt3 expression in OCCM‐30 cells (Figure S3). After osteogenic induction, decreased mRNA and protein expression of mineralisation‐related markers were detected in 3‐TYP‐treated OCCM‐30 cells (Figure S4A,B). The ALP activity and mineralised nodule formation capacity were also impaired by 3‐TYP (Figure S4C,D). Further, Sirt3‐silenced cells were constructed by lentivirus infection (Figure S5) and successfully identified (Figure 2F,G). Similarly, weakened mineralisation of shSirt3 cells compared with shNC cells was measured by RT‐qPCR (Figure 2H), western blot (Figure 2I), ALP staining (Figure 2J) and ARS (Figure 2K). Besides, a higher ROS level in shSirt3 cells was detected, which could partially be responsible for the mineralisation reduction (Figure 2L). Therefore, Sirt3 serves as an indispensable regulator maintaining cementum homeostasis.

### 3.3 Sirt3 Activation by HKL Rescues *Porphyromonas gingivalis* ‐Suppressed Cementoblast Mineralisation

**FIGURE 3:**
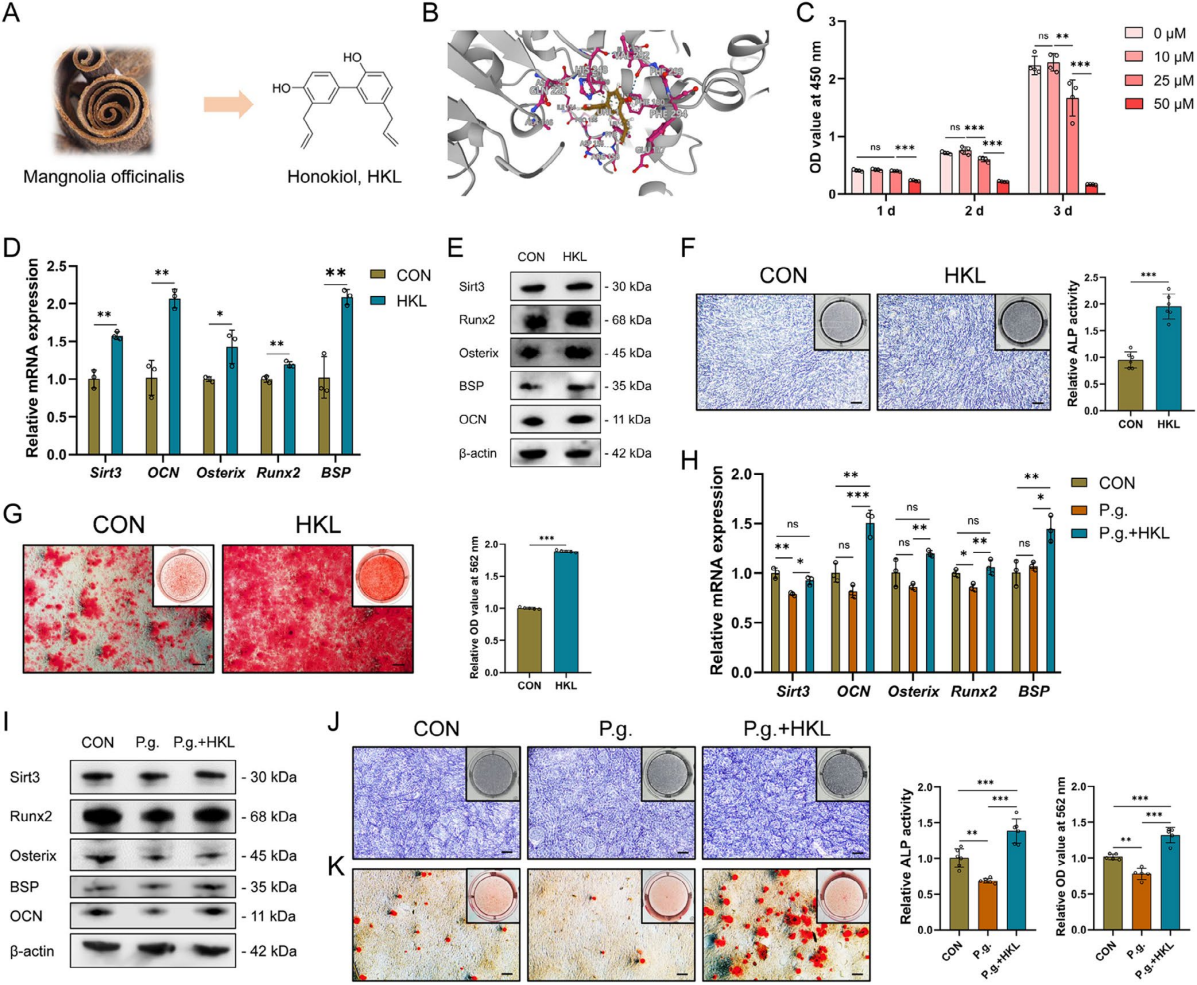
Sirt3 activation by HKL rescues P.g.‐suppressed cementoblast mineralisation. Origin and chemical structure of HKL (A). Molecule docking results between HKL and Sirt3 (B). Cytotoxicity of HKL to OCCM‐30 cells was measured by the CCK‐8 assay (C). The mRNA and protein expression of Sirt3 and mineralisation‐related markers in OCCM‐30 cells induced with or without HKL for 2 days was detected by RT‐qPCR (D) and western blot (E), respectively. ALP activity of OCCM‐30 cells induced with or without HKL for 7 days was measured by ALP staining (F). Mineralised nodule formation capacity of OCCM‐30 cells induced with or without HKL for 14 days was measured by ARS (G). The rescuing effect of HKL on the expression of Sirt3 and mineralisation‐related markers suppressed by P.g. (MOI = 100) in OCCM‐30 cells induced for 2 days was verified by RT‐qPCR (H) and western blot (I). The rescuing effect of HKL on the ALP activity suppressed by P.g. (MOI = 100) in OCCM‐30 cells induced for 7 days was verified by ALP staining (J). The rescuing effect of HKL on the mineralised nodule formation capacity suppressed by P.g. (MOI = 100) in OCCM‐30 cells induced for 10 days was verified by ARS (K). CCK‐8, cell counting kit‐8; HKL, honokiol. *p < 0.05, **p < 0.01 and ***p < 0.001. *p* > 0.05 was considered not significant (ns).

HKL, a natural pleiotropic small molecule isolated from *Magnolia officinalis*, was applied for Sirt3 activation (Figure 3A). Our molecular docking results uncovered that HKL could bind to Sirt3 at PHE180 (Pi stacking), HIS248 (Pi stacking) and VAL292 (hydrogen bond) with a binding energy of −8.673 kcal/mol (Figure 3B). By the CCK‐8 assay, a concentration of 10 μM HKL was selected for cell stimulation (Figure 3C). RT‐qPCR and western blot experiments confirmed the upregulation of Sirt3 in OCCM‐30 cells by HKL (Figure 3D,E). Meanwhile, increased mRNA and protein expression of mineralisation‐related markers, ALP activity and mineralised nodule formation capacity were detected (Figure 3D–G), indicating that Sirt3 activation by HKL facilitated CB mineralisation.

Further, we wonder whether Sirt3 activation could rescue P.g.‐inhibited cell mineralisation. The results showed that Sirt3 downregulation in P.g.‐infected cells was reversed after HKL treatment (Figure 3H,I). Also, P.g.‐suppressed CB mineralisation was totally rescued by HKL (Figure 3H–K). Thus, these results indicated that Sirt3 mediated P.g.‐suppressed CB mineralisation. Targeting Sirt3 by HKL shows an outstanding mineralisation‐rescuing effect.

### 3.4 Sirt3/SOD2 Axis Mediates Cementoblast Mineralisation

**FIGURE 4:**
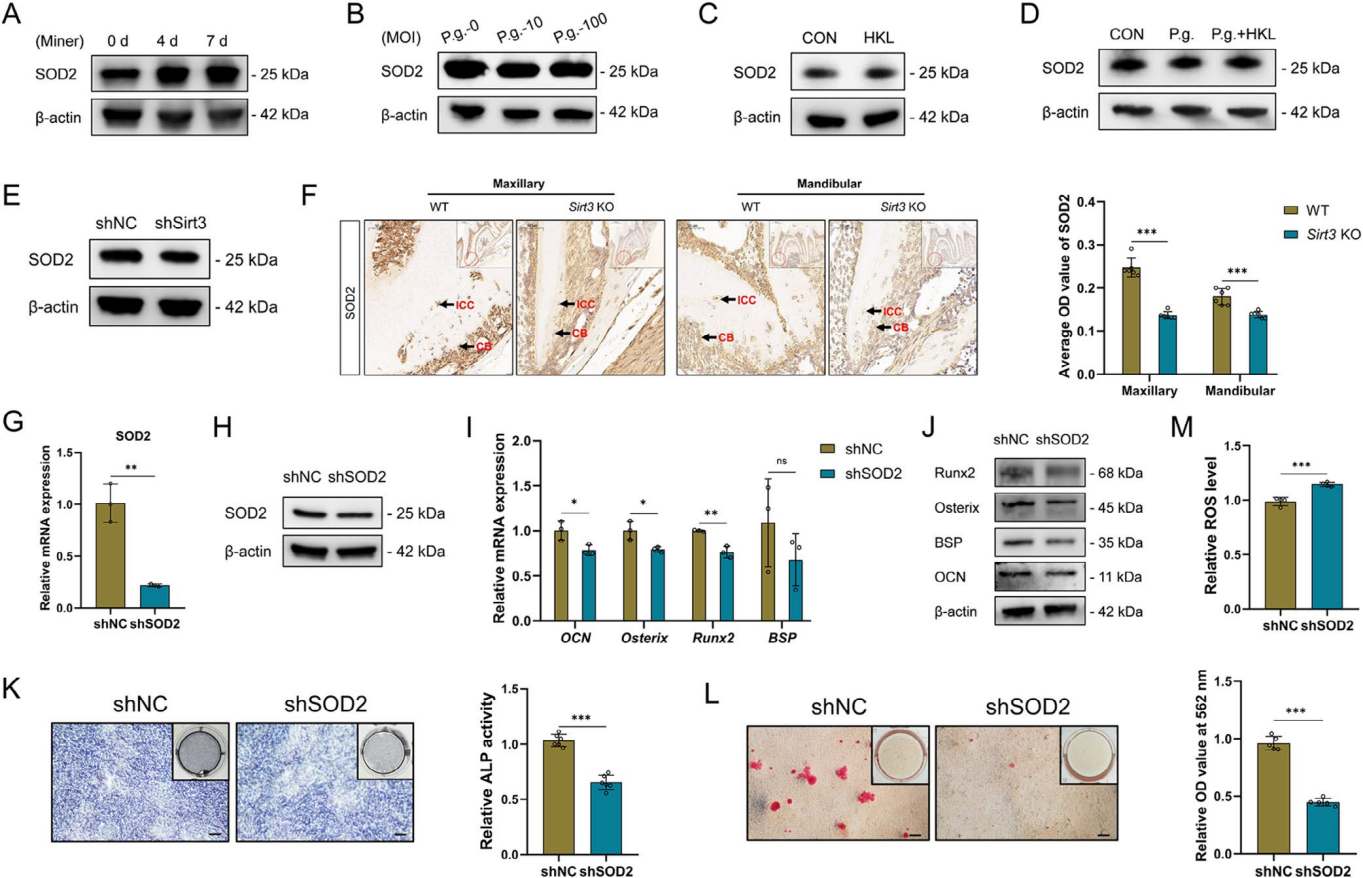
Sirt3/SOD2 axis mediates cementoblast mineralisation. The expression of SOD2 in OCCM‐30 cells induced for 0, 4 and 7 days was detected by western blot (A). The expression of SOD2 in OCCM‐30 cells infected with different MOIs of P.g. (0, 10 and 100) for 1 day was detected by western blot (B). The expression of SOD2 in OCCM‐30 cells induced with or without HKL for 2 days was detected by western blot (C). The rescuing effect of HKL on the expression of SOD2 suppressed by P.g. (MOI = 100) in OCCM‐30 cells induced for 2 days was verified by western blot (D). The expression of SOD2 in shNC and shSirt3 cells was verified by western blot (E). The expression of SOD2 in cementoblasts of the first molars from WT and *Sirt3* KO mice (F). The expression of SOD2 in SOD2‐silenced OCCM‐30 cells (shSOD2) and the control cells (shNC) was verified by RT‐qPCR (G) and western blot (H). The mRNA and protein expression of mineralisation‐related markers in shNC and shSOD2 cells induced for 2 days was detected by RT‐qPCR (I) and western blot (J), respectively. ALP activity of shNC and shSOD2 cells induced for 7 days was measured by ALP staining (K). Mineralised nodule formation capacity of shNC and shSOD2 cells induced for 10 days was measured by ARS (L). Intracellular ROS level in shNC and shSOD2 cells was determined by a ROS assay kit (M). SOD2, superoxide dismutase 2. *p < 0.05, **p < 0.01 and ***p < 0.001. *p* > 0.05 was considered not significant (ns).

SOD2 is a classical downstream molecule of Sirt3. We then focused on the expression pattern and mineralisation‐regulatory effect of SOD2 on CB mineralisation. Similar to Sirt3, the expression of SOD2 in OCCM‐30 cells increased gradually with the extension of mineralisation days (Figure 4A), while it decreased slightly under P.g. infection (Figure 4B). The Sirt3 activator HKL was found to upregulate the SOD2 expression and also rescue the P.g.‐suppressed SOD2 expression in OCCM‐30 cells (Figure 4C,D). When the upstream Sirt3 was silenced, the expression of SOD2 in OCCM‐30 cells was detected to be slightly downregulated (Figure 4E). A similar trend was also confirmed in CB s of *Sirt3* KO mice (Figure 4F).

To figure out the involvement of SOD2 in CB mineralisation, SOD2‐silenced cells were constructed by lentivirus infection (Figure S6) and identified successfully (Figure 4G,H). Decreased expression of mineralisation‐related markers, ALP activity and mineralised nodule formation capacity of shSOD2 cells compared with shNC cells were demonstrated by RT‐qPCR (Figure 4I), western blot (Figure 4J), ALP staining (Figure 4K) and ARS (Figure 4L), respectively. Accordingly, an increased intracellular ROS level in OCCM‐30 cells was detected when the key free radical scavenger SOD2 was knocked down (Figure 4M). Therefore, the mineralisation‐promoting SOD2 works as a downstream regulator during CB mineralisation.

### 3.5 Sirt3 Deacetylates SOD2 Through K68 to Orchestrate Cementoblast Mineralisation

**FIGURE 5:**
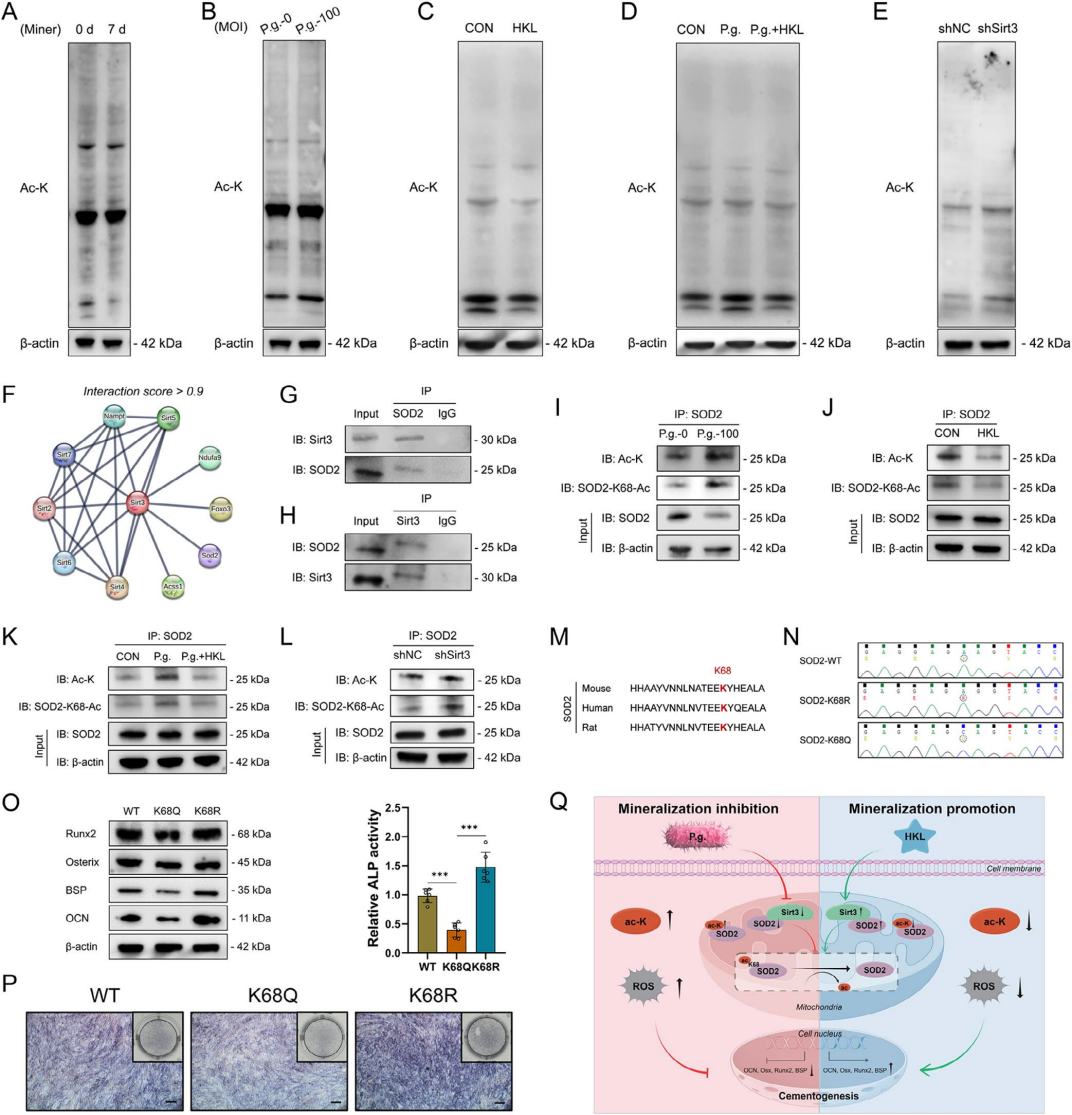
Sirt3 deacetylates SOD2 through K68 to orchestrate cementoblast mineralisation. The pan acetylation in OCCM‐30 cells induced for 0 and 7 days was detected by western blot (A). The pan acetylation in OCCM‐30 cells infected with P.g. (MOI = 100) for 1 day was detected by western blot (B). The pan acetylation in OCCM‐30 cells induced with HKL for 2 days was detected by western blot (C). The reversing effect of HKL on the pan acetylation increased by P.g. (MOI = 100) in OCCM‐30 cells induced for 2 days was verified by western blot (D). The pan acetylation in shNC and shSirt3 cells was verified by western blot (E). The interacting proteins of Sirt3 with an interaction score over 0.9 were mapped by the STRING database (F). The interaction between Sirt3 and SOD2 in OCCM‐30 cells was verified by co‐IP (G, H). The overall acetylation of SOD2 and specific K68 acetylation in OCCM‐30 cells infected with P.g. (MOI = 100) for 1 day (I) and induced with HKL for 2 days (J) was detected by co‐IP. The reversing effect of HKL on the overall acetylation of SOD2 and specific K68 acetylation increased by P.g. (MOI = 100) in OCCM‐30 cells induced for 2 days was verified by co‐IP (K). The overall acetylation of SOD2 and specific K68 acetylation in shNC and shSirt3 cells was verified by co‐IP (L). The conservation of SOD2‐K68 from mouse, rat and human was confirmed by NCBI database (M). DNA sequencing results of SOD2‐WT, and the mutated SOD2‐K68R, SOD2‐K68Q plasmids (N). The expression of mineralisation‐related markers in WT, K68Q and K68R plasmid‐transfected OCCM‐30 cells induced for 2 days was detected by western blot (O). The ALP activity of WT, K68Q and K68R plasmid‐transfected OCCM‐30 cells induced for 7 days was measured by ALP staining (P). Schematic diagram of the study (Q). Co‐IP, co‐immunoprecipitation; K, lysine; Q, glutamine; R, arginine. ***p < 0.001.

Due to the role of Sirt3 at the post‐translational level, the pan acetylation under different conditions was then detected. Western blot results showed that pan acetylation decreased in mineralised cells (Figure 5A), while it increased in P.g.‐stimulated cells (Figure 5B). Consistent with this, the mineralisation‐facilitating drug HKL suppressed the pan acetylation (Figure 5C) and also relieved the P.g.‐induced pan acetylation in OCCM‐30 cells (Figure 5D). Without doubt, the pan acetylation level in Sirt3‐silenced OCCM‐30 cells (Figure 5E) and CBs of *Sirt3* KO mice (Figure S7) was also upregulated. The above results indicated a negative correlation between pan acetylation and cell mineralisation.

Further, the interacting proteins of Sirt3 with an interaction score over 0.9 were mapped by the STRING database, among which the above‐mentioned regulator SOD2 was selected (Figure 5F). Co‐IP experiments verified the subcellular interaction between Sirt3 and SOD2 in OCCM‐30 cells (Figure 5G,H), indicating that downstream SOD2 might be the potential deacetylation protein of Sirt3. IP results revealed that the SOD2 acetylation and specific SOD2‐K68 acetylation increased under P.g. infection (Figure 5I) and decreased under HKL induction in OCCM‐30 cells (Figure 5J). HKL also reversed the SOD2 acetylation and K68 acetylation upregulated by P.g. (Figure 5K). Increased overall SOD2 acetylation and K68 acetylation in Sirt3‐silenced OCCM‐30 cells were demonstrated (Figure 5L).

Conservation of SOD2‐K68 from mouse, rat and human was confirmed by the NCBI database (Figure 5M). The regulatory effect of SOD2‐K68 on CB mineralisation was then studied. 68‐K of SOD2 was mutated to Q to simulate acetylation and mutated to R to simulate deacetylation (Figure 5N). After induction, decreased expression of mineralisation‐related markers and ALP activity in the K68Q group and increased expression of mineralisation‐related markers and ALP activity in the K68R group were confirmed by western blot (Figure 5O) and ALP staining (Figure 5P). Altogether, Sirt3 deacetylates SOD2‐K68 to facilitate CB mineralisation.

## 4 Discussion

In all, this study focused on the role of Sirt3 in cementogenesis and uncovered the involvement of the Sirt3/SOD2 axis in P.g.‐perturbed cementoblast mineralisation. As shown in Figure 5Q, Sirt3 was found to be increased during cementogenesis and decreased under P.g.‐elicited inflammation. Sirt3 was demonstrated to regulate cementogenesis positively. Reestablishing the Sirt3/SOD2 axis by HKL rescued P.g.‐suppressed CB mineralisation. Mechanistically, Sirt3 deacetylated SOD2 via K68 to mediate cementoblast mineralisation under P.g. infection.

Cementum can be defined as the extracellular matrix (ECM) comprising Sharpey's fibrils, collagen, proteoglycans, glycosaminoglycans and hydroxyapatite [2]. It is an anatomical structure for periodontal ligament attachment and is highly similar to other hard tissues such as bone and dentin in terms of the structure, composition and mineralisation process [24, 25]. Also, CBs embedded in the ECM and the cementocytes that originate from them share similarities with osteocytes [26]. Therefore, the regulating genes attributed to osteogenesis and dentinogenesis may be appropriate for cementogenesis. Since it was reported that Sirt3 was a positive regulator required for the differentiation of osteogenic lineage cells, such as bone marrow mesenchymal stem cells, MC3T3‐E1 cells and osteoblasts [13, 27, 28], and decreased bone mass and an increased number of osteoclasts were found in Sirt3^−/−^ mice [13, 29], we guessed Sirt3 might also regulate cementogenesis positively, and lower cementum volume in Sirt3^−/−^ mice compared with the control mice might be observed. Our results confirmed the above speculation, showing decreased cementogenesis in Sirt3^−/−^ mice, 3‐TYP‐treated and Sirt3‐silenced CBs, and increased CB mineralisation in Sirt3‐activated CBs. Besides, the regulatory effect of Sirt3 on dentinogenesis has not been studied yet. Based on HE results, we suppose Sirt3 may also facilitate dentinogenesis and the dentinogenic differentiation of dental pulp stem cells.

Cementum resorption occurs in patients with periodontitis and apical periodontitis, in which the gram‐negative oral anaerobe P.g. is the keystone pathogen [30]. CBs lining the surface of tooth roots are sensitive to P.g. infection [8]. In our previous work, casein kinase 2 interacting protein‐1 (Ckip‐1) was demonstrated to negatively mediate P.g.‐suppressed CB mineralisation [8]. In the present study, we first verified the positive‐regulating role of Sirt3 in cementogenesis. Further, Sirt3 was proved to mediate P.g.‐stimulated CB mineralisation, and Sirt3 activation rescued CB mineralisation suppressed by P.g. Thus, these works are essential for understanding the balanced signalling network involved in cementogenesis.

Sirt3 has been well‐documented to deacetylate various downstream mitochondria‐related proteins, such as cytochrome c oxidase subunit 4 isoform 2 (COX4I2), mitochondrial transcription factor A (TFAM) and peroxiredoxin 3 (PRDX3), to regulate mitochondrial homeostasis partially through ROS production [31, 32, 33]. SOD2 is a mitochondrial regulator of ROS and is reported to be deacetylated by Sirt3 in osteoblasts, endothelial progenitor cells and myocardial cells [13, 34, 35]. In our study, we demonstrated that Sirt3 could bind and deacetylate SOD2 in CBs to mediate the mineralisation process with or without P.g. infection. Of note, decreased expression of SOD2 and reduced ROS production were found when Sirt3 was knocked down, which was consistent with the results in Sirt3‐silenced MC3T3‐E1 cells [27]. However, the expression of SOD2 remained unchanged in Sirt3‐silenced osteoblasts, though the SOD2 activity was suppressed [13]. We hypothesised that this inconformity was due to different cell types, which implied that the osteogenic precursor MC3T3‐E1 cells were more like CBs than osteoblasts. Also, the expression pattern of SOD2 in Sirt3‐silenced cementocytes may be in line with the terminally differentiated osteoblasts. Further, K68 was detected to be the deacetylation site of SOD2 by Sirt3 in CBs. Whether other sites like K122 mediate CB mineralisation, and which one dominates the process, needs further exploration [36].

In terms of therapy, HKL was applied to reestablish the Sirt3/SOD2 axis suppressed by P.g. We confirmed that HKL could bind to Sirt3 with a binding energy of −8.673 kcal/mol. The simulation results were better than previously reported [32]. Besides HKL, other small molecule drugs have also been used to activate Sirt3, such as ganoderic acid D, resveratrol and kaempferol [37, 38, 39]. In this study, HKL was demonstrated to reduce the acetylation level and rescue P.g.‐suppressed CB mineralisation; however, much work remains to be done in the selection of small molecule drugs for the treatment of P.g.‐perturbed cementogenesis. On the other hand, K68 of SOD2 was proved to be a therapeutic target for cementum resorption. The results showed that the specific K68 acetylation increased as a result of Sirt3 suppression after P.g. infection. When 68‐lysine (K) of SOD2 was mutated to arginine (R) to simulate deacetylation, increased mineralisation was observed in CBs.

## 5 Conclusions

In this study, the facilitating role of Sirt3 in cementogenesis was revealed for the first time. Sirt3 was demonstrated to deacetylate SOD2 via K68 to orchestrate CB mineralisation under P.g. infection. Reestablishment of the Sirt3/SOD2 axis by HKL reduced the acetylation of SOD2, thus rescuing P.g.‐suppressed CB mineralisation. Altogether, our study provides a novel target and a specific therapeutic drug for the restoration of P.g.‐damaged cementum.

## Author Contributions

**Xin Huang:** conceptualisation, data curation, formal analysis, funding acquisition, investigation, methodology, supervision, writing – original draft, writing – review and editing. **Huiqing Gou:** data curation, formal analysis, funding acquisition, investigation, methodology and writing – original draft. **Jirong Xie:** data curation, formal analysis, investigation, methodology and writing – original draft. **Yonglin Guo** and **Yifei Deng:** data curation, investigation and methodology. **Yan Xu** and **Zhengguo Cao:** conceptualisation, funding acquisition, resources, supervision, visualisation, writing – review and editing.

## Conflicts of Interest

The authors declare no conflicts of interest.

## Supporting information


**Data S1.** Supporting Information.

## Data Availability

The data are available from the corresponding author on reasonable request.
