# Supplementary material for: Sirt3 Rescues Porphyromonas gingivalis‐Impaired Cementogenesis via SOD2 Deacetylation
Source: Cell Prolif. 2025 Mar 11;58(9):e70022. doi: 10.1111/cpr.70022 (PMC12414636; doi:10.1111/cpr.70022)
Supplement: Supplementary file 1 — Data S1. Supporting Information. [file CPR-58-e70022-s001.docx]

**Sirt3 rescues P.g.-impaired cementogenesis via SOD2 deacetylation**

**1. Supplementary figures**

*
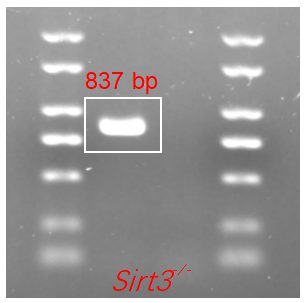
*

**Figure S1**. Sirt3*^-/-^* mice genotypes were identified by agarose gel electrophoresis. The homozygous genotype is represented by a single band of 837 bp. The DNA marker used in this study is DL2000.


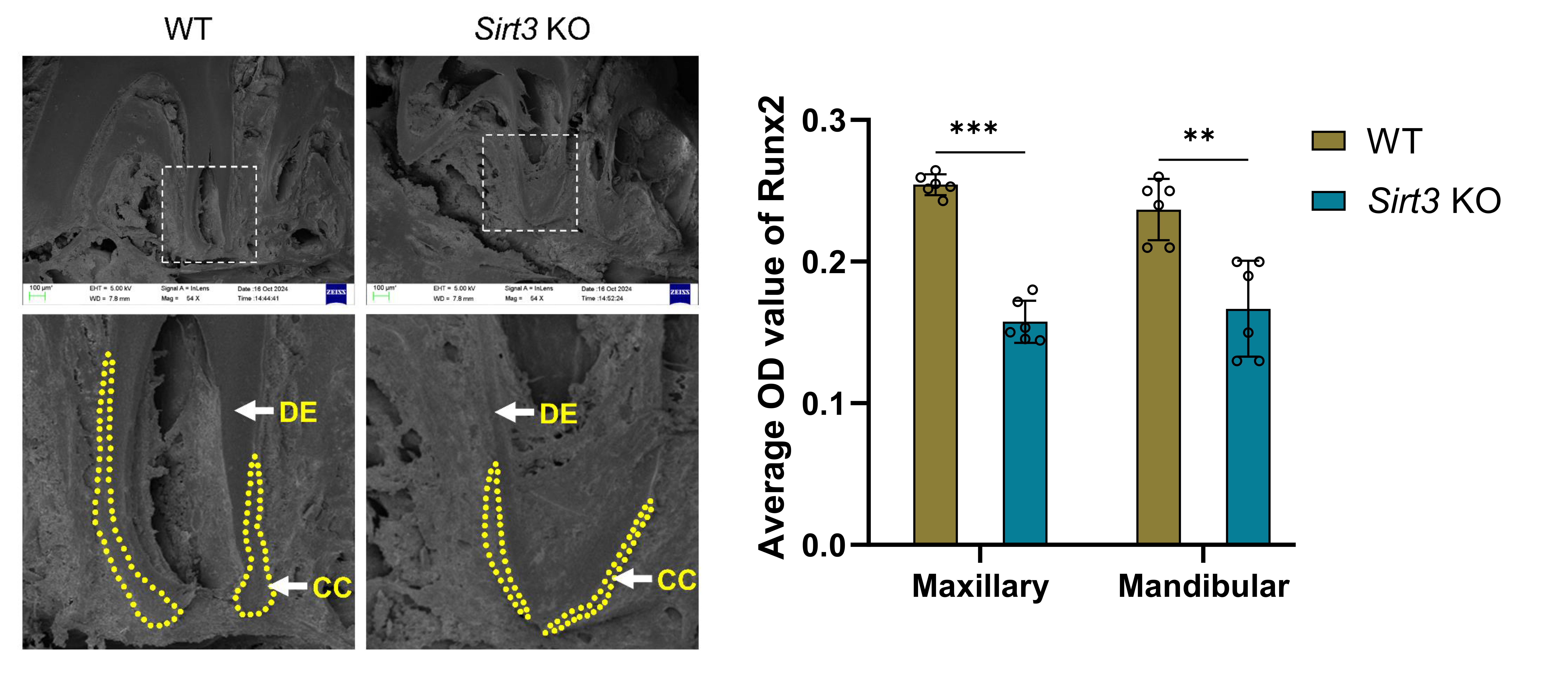


**Figure S2**. SEM images of cellular cementum of the distal root of mandibular first molars of WT mice and *Sirt3* KO mice.


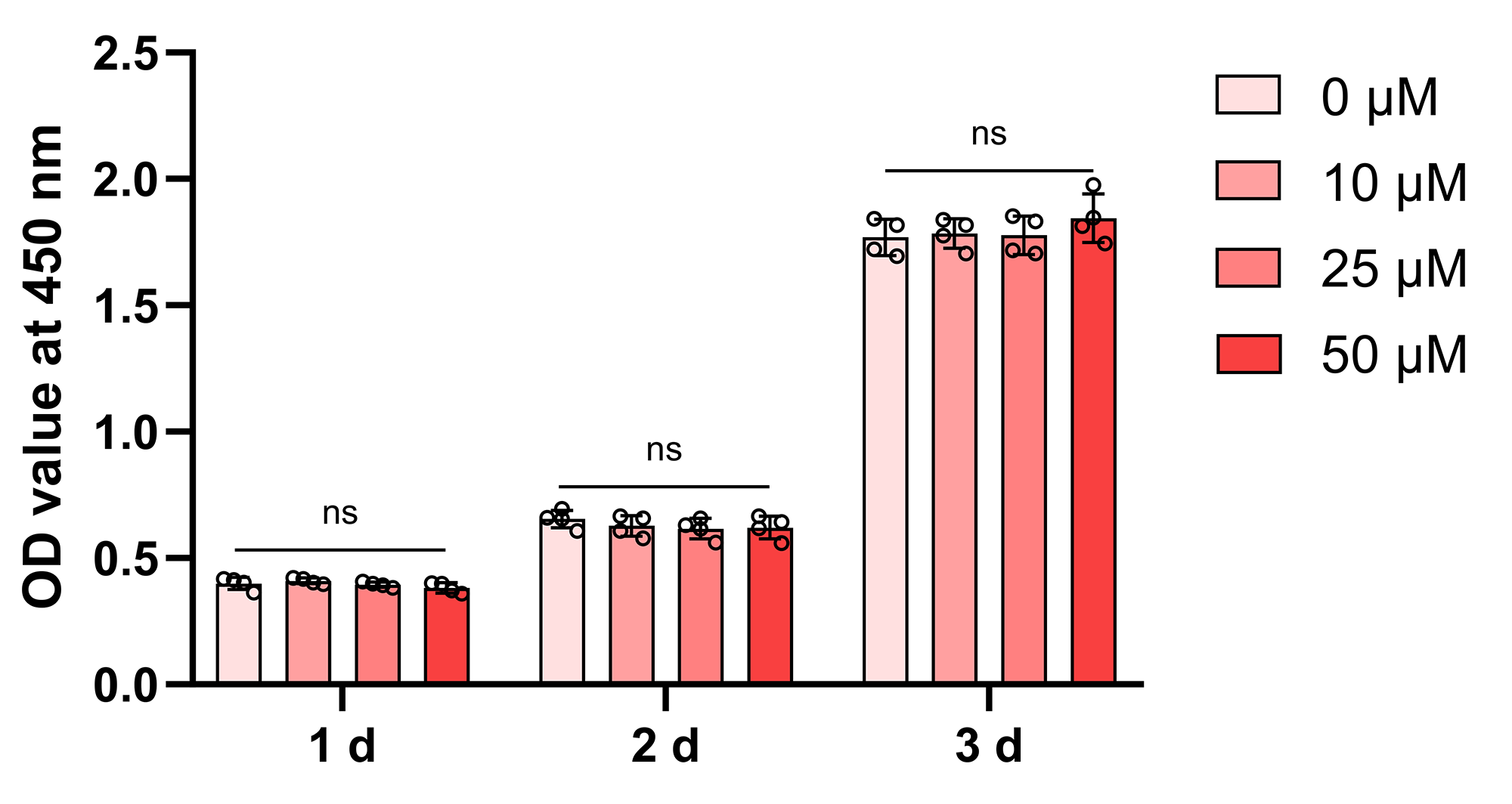


**Figure S3**. Cytotoxicity of 3-TYP to OCCM-30 cells was measured by the CCK-8 assay. *P*>0.05 was considered not significant (ns).


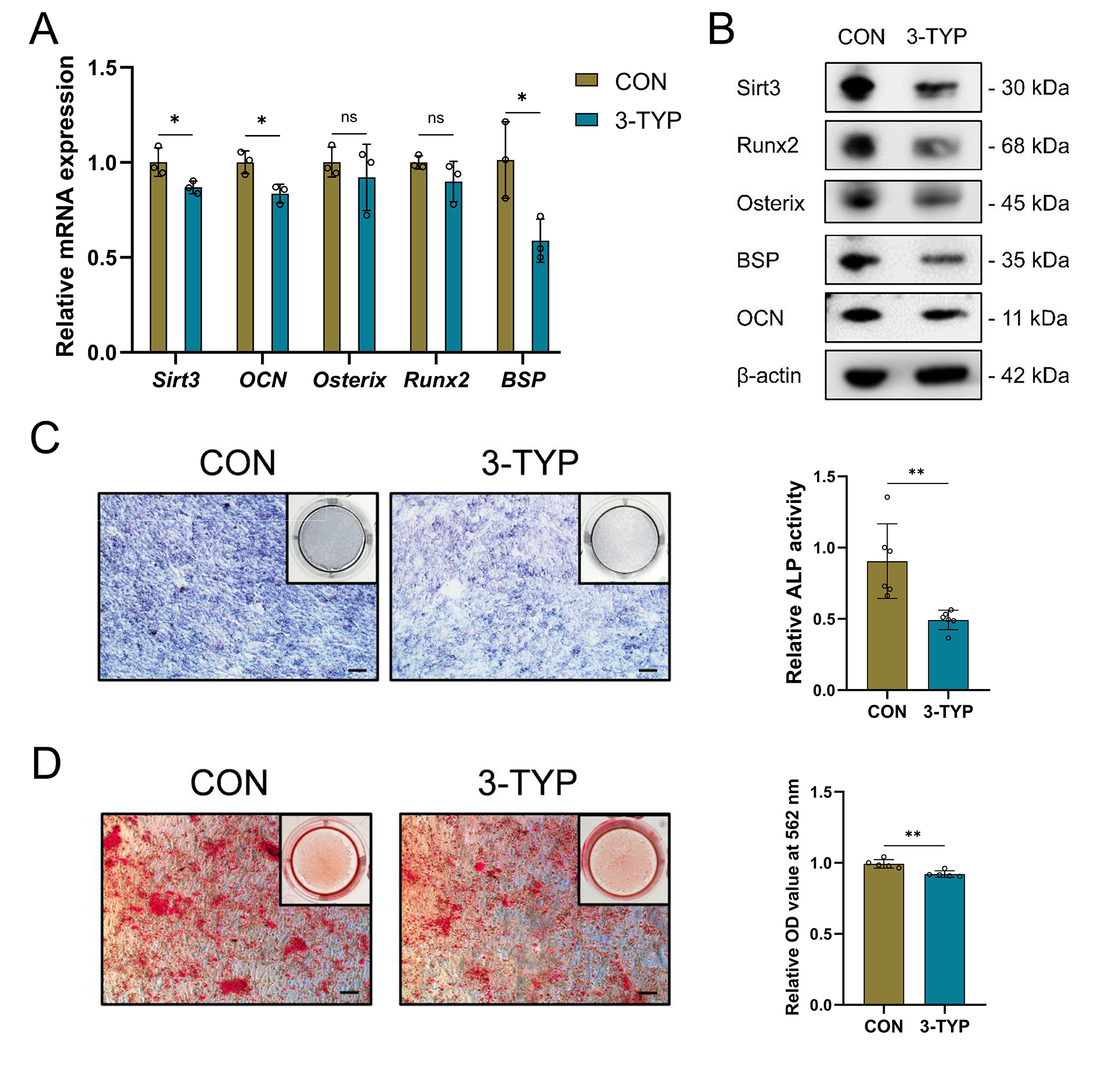


**Figure S4**. Sirt3 suppression by 3-TYP impairs cementoblast mineralization. The mRNA and protein expression of Sirt3 and mineralization-related markers in OCCM-30 cells induced with or without 3-TYP for 2 days was detected by RT-qPCR (A) and western blot (B), respectively. ALP activity of OCCM-30 cells induced with or without 3-TYP for 7 days was measured by ALP staining (C). Mineralized nodule formation capacity of OCCM-30 cells induced with or without 3-TYP for 14 days was measured by ARS (D). **P*<0.05, ***P*<0.01. *P*>0.05 was considered not significant (ns).


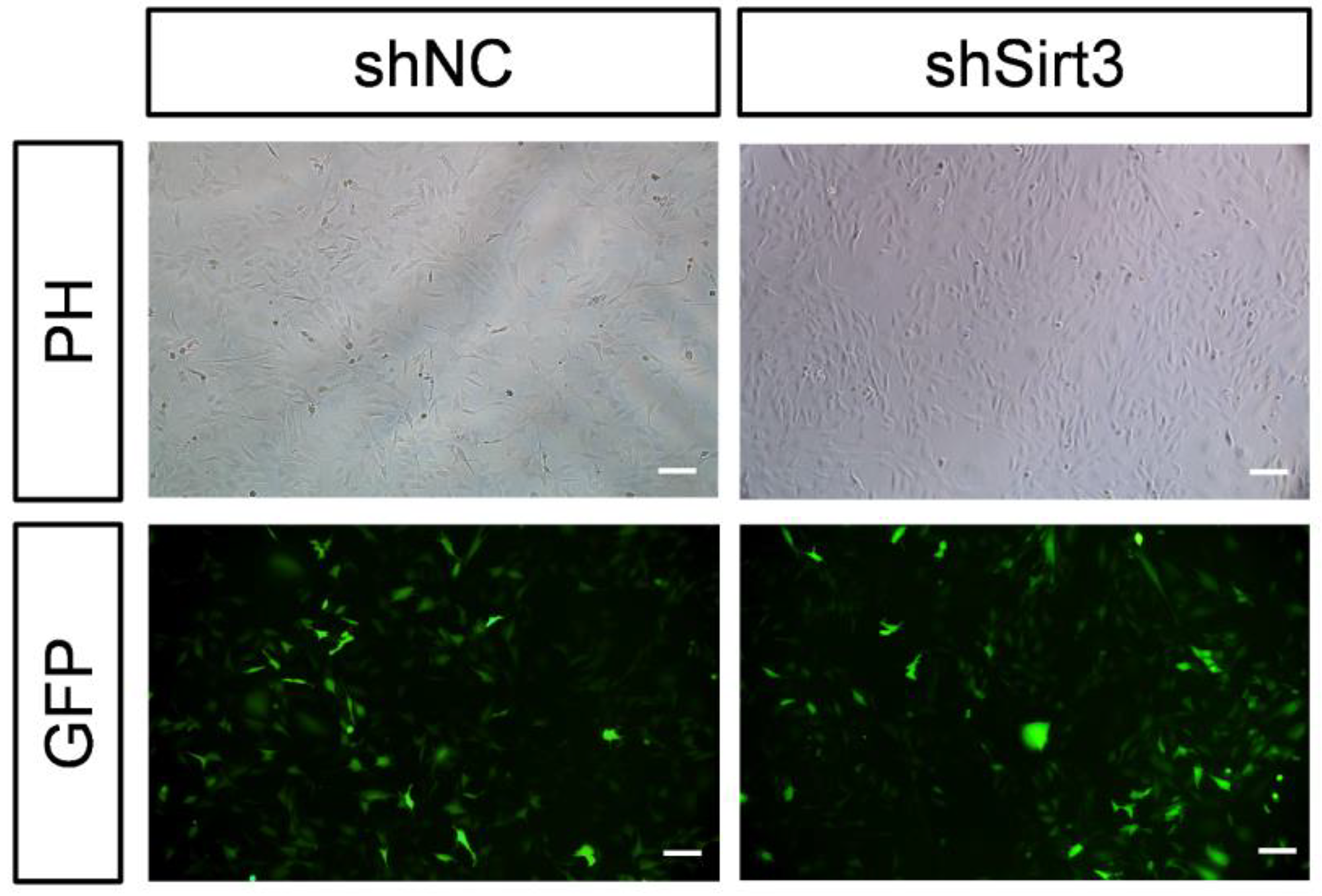


**Figure S5**. Sirt3-silenced cells (shSirt3) and the control cells (shNC) were constructed by lentivirus infection, and the green fluorescence was observed to identify the transfection efficiency. PH, phase contrast; GFP: green fluorescence protein.


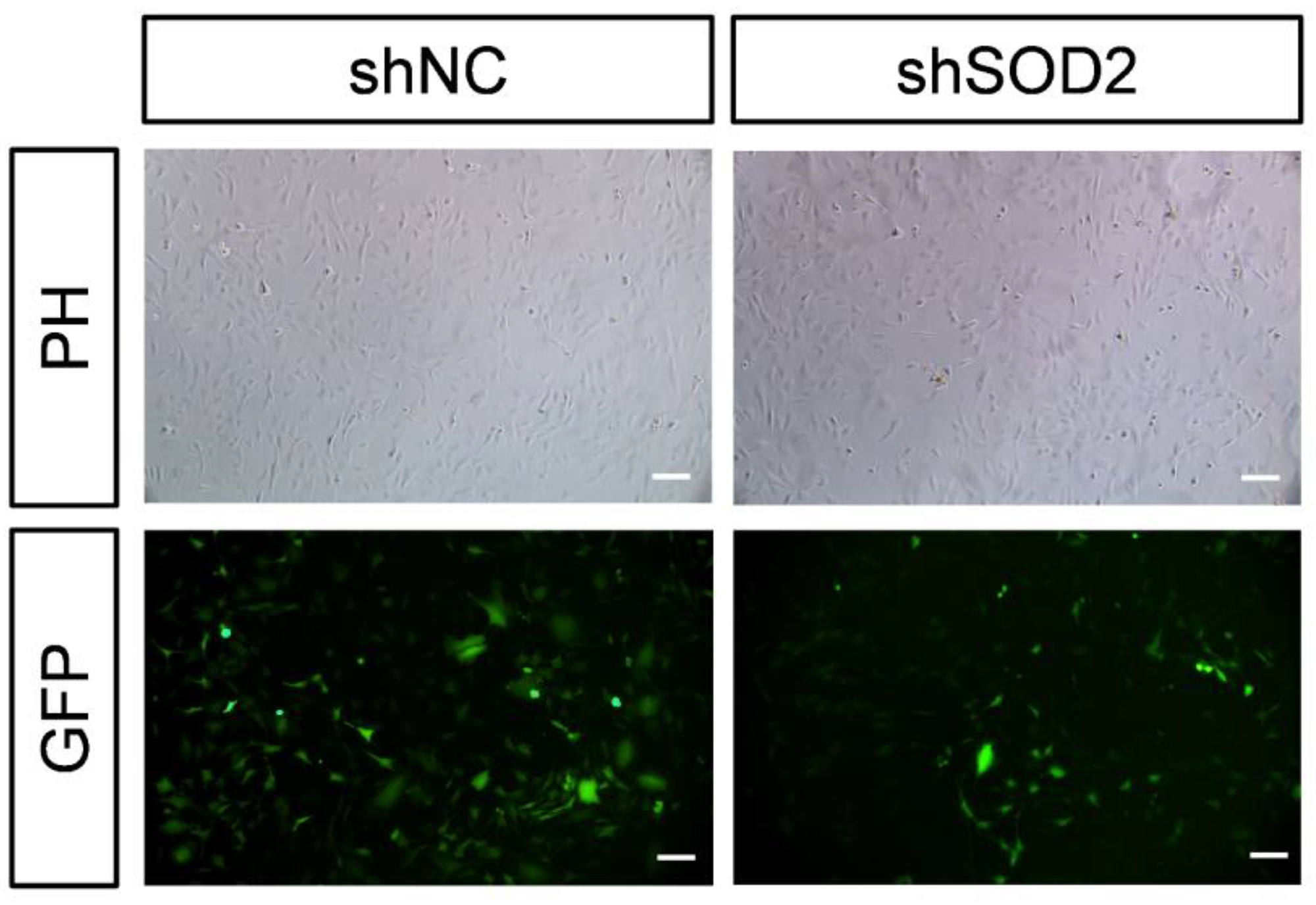


**Figure S6**. SOD2-silenced cells (shSOD2) and the control cells (shNC) were constructed by lentivirus infection, and the green fluorescence was observed to identify the transfection efficiency.


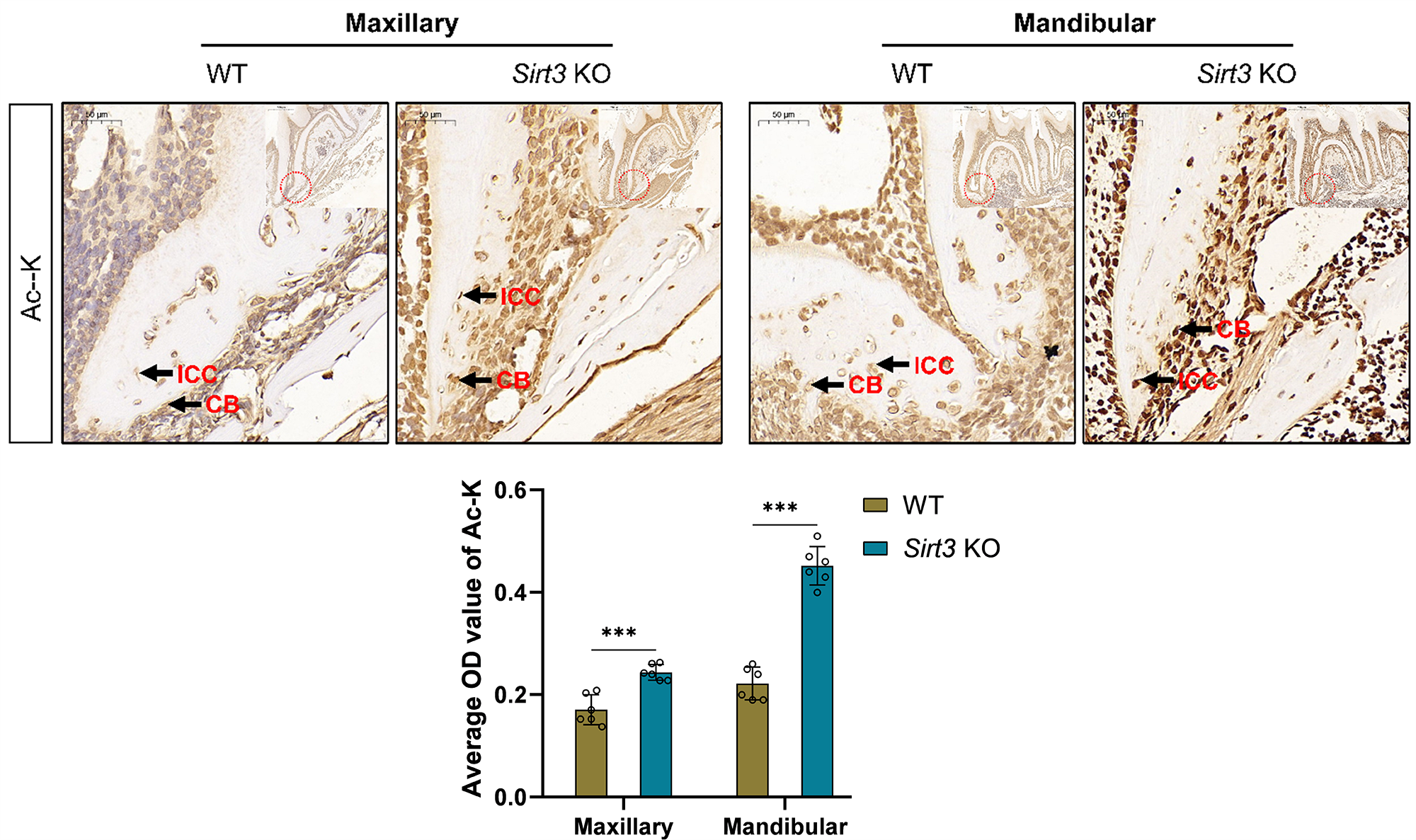


**Figure S7**. Pan acetylation level of cementoblasts of *Sirt3* KO mice and WT mice was determined by IHC.

**2. Supplementary tables**

**Table S1:** Primers used for RT-qPCR

| Gene | Primer sequences (5’-3’) |
| --- | --- |
| *OCN* | F-TGAACAGACTCCGGCGCTAC  R-AGGGCAGCACAGGTCCTAA |
| *Osterix* | F-CCTCGCTCTCTCCTATTGCAT  R-GTTGAGGAGGTCGGAGCATA |
| *Runx2* | F-CCAGGCAGGTGCTTCAGAACTG  R-ACATGCCGAGGGACATGCCTG |
| *BSP* | F-GAGCCTCGTGGCGACACTTA  R-AATTCTGACCCTCGTAGCCTTCATA |
| *Sirt1* | F-AGAACCACCAAAGCGGAAA  R-TCCCACAGGAGACAGAAACC |
| *Sirt2* | F-CAAGCCAACCATCTGCCACTA  R-CCCGCCACTCGTTCCA |
| *Sirt3* | F-TGCTACTCATCTTGGGACCT  R-CACCAGCCTTTCCACACC |
| *Sirt4* | F-GTCCCGTGCTGTGATCGA  R-CGGGCGGTGAGGATGAAC |
| *Sirt5* | F-GCCTCCCCACAAAGCAAGA  R-AACCCCACTCTCCGCACTAA |
| *Sirt6* | F-CCTGCCCCTTGCCACTAA  R-GCACATCACCTCATCCACGTA |
| *Sirt7* | F-GCCAGGAGGAGGTGTGTGA  R-GGCTCCGCTTCGCTTAGGT |
| *IL-6* | F-TGTGCAATGGCAATTCTGAT  R-CTCTGAAGGACTCTGGCTTTG |
| *Ptgs2* | F-TTCAACACACTCTATCACTGGC  R-AGAAGCGTTTGCGGTACTCAT |
| *SOD2* | F-GCAGTGTGCGGCACCAGCAG  R-TCCCTTGGCCAACGCCTCCT |
| *β-actin* | F-GATTACTGCTCTGGCTCCT  R-TGGAAGGTGGACAGTGAG |

F, forward; R, reverse; *OCN*, osteocalcin; *Runx2*, RUNX family transcription factor 2; *BSP*, sialoprotein; *Sirt1~7*, sirtuin 1~7; *IL-6*, interleukin-6; *Ptgs2*, prostaglandin endoperoxide synthase 2; *SOD2*, superoxide dismutase 2.

**Table S2**: Antibodies used in this study for IB

| Antibody | Brand | Dilution rate |
| --- | --- | --- |
| OCN | sc-390877, Santa Cruz, USA | 1:250 |
| Osterix | ab22552, Abcam, UK | 1:1000 |
| Runx2 | ab236639, Abcam, UK | 1:1000 |
| BSP | AF0227, Affinity, USA | 1:1000 |
| Sirt3 | A20805, ABclonal, Wuhan, China | 1:1000 |
| IL-6 | 21865-1-AP, Proteintech, USA | 1:800 |
| IL-1β | A16288, ABclonal, Wuhan, China | 1:1000 |
| SOD2 | 24127-1-AP, Proteintech, USA | 1:5000 |
| Acetyl-lysine | DF7729, Affinity, USA | 1:1000 |
| Acetyl-SOD2-Lys68 | AF3751, Affinity, USA | 1:1000 |
| β-actin | 66009-1-AP, Proteintech, USA | 1:15000 |
| Goat anti-rabbit IgG | SA00001-2, Proteintech, USA | 1:8000 |
| Goat anti-mouse IgG | SA00001-1, Proteintech, USA | 1:10000 |

Ac-K, Acetyl-lysine; SOD2-K68-Ac, Acetyl-SOD2 (Lys68).

**Table S3**: Antibodies used in this study for IHC or IF

| Antibody | Brand | Dilution rate |
| --- | --- | --- |
| Runx2 | ab236639, Abcam, UK | 1:200 |
| OCN | sc-390877, Santa Cruz, USA | 1:50 |
| Acetyl-lysine | DF7729, Affinity, USA | 1:200 |
| Sirt3 | A20805, ABclonal, Wuhan, China | 1:200 |
| SOD2 | 24127-1-AP, Proteintech, USA | 1:200 |
| Porphyromonas gingivalis | 60BG1.3, DSHB, USA | 5 μg/mL |
| Goat anti-mouse IgG-FITC | AS001, ABclonal, Wuhan, China | 1:200 |
| Goat anti-rabbit IgG-Cy3 | AS007, ABclonal, Wuhan, China | 1:200 |

**Table S4**: Targeting sequences used for shRNA construction

| Gene | Targeting sequences (5’-3’) |
| --- | --- |
| *Sirt3* | GCCCAATGTCACTCACTACTT |
| *SOD2* | GCTTACTACCTTCAGTATAAA |
